# Critical assessment and ramifications of a purported marine trophic cascade

**DOI:** 10.1038/srep20970

**Published:** 2016-02-15

**Authors:** R. Dean Grubbs, John K. Carlson, Jason G. Romine, Tobey H. Curtis, W. David McElroy, Camilla T. McCandless, Charles F. Cotton, John A. Musick

**Affiliations:** 1 Florida State University Coastal and Marine Laboratory, 3618 Hwy 98, St. Teresa, FL 32358, USA; 2 NOAA/National Marine Fisheries Service, Southeast Fisheries Science Center, 3500 Delwood Beach Road, Panama City, FL 32408, USA; 3 U.S. Geological Survey, Western Fisheries Research Center, Columbia River Research Laboratory, Cook, WA 98605, USA; 4 NOAA/National Marine Fisheries Service, Greater Atlantic Regional Fisheries Office, 55 Great Republic Drive, Gloucester, Massachusetts, 01930, USA; 5 NOAA/National Marine Fisheries Service, Northeast Fisheries Science Center, 166 Water Street, Woods Hole, MA 02543, USA; 6 NOAA/National Marine Fisheries Service, Northeast Fisheries Science Center, 28 Tarzwell Drive, Narragansett, RI 02882, USA; 7 Virginia Institute of Marine Science, 1208 Greate Rd., Gloucester Point, VA 32062, USA

## Abstract

When identifying potential trophic cascades, it is important to clearly establish the trophic linkages between predators and prey with respect to temporal abundance, demographics, distribution, and diet. In the northwest Atlantic Ocean, the depletion of large coastal sharks was thought to trigger a trophic cascade whereby predation release resulted in increased cownose ray abundance, which then caused increased predation on and subsequent collapse of commercial bivalve stocks. These claims were used to justify the development of a predator-control fishery for cownose rays, the “Save the Bay, Eat a Ray” fishery, to reduce predation on commercial bivalves. A reexamination of data suggests declines in large coastal sharks did not coincide with purported rapid increases in cownose ray abundance. Likewise, the increase in cownose ray abundance did not coincide with declines in commercial bivalves. The lack of temporal correlations coupled with published diet data suggest the purported trophic cascade is lacking the empirical linkages required of a trophic cascade. Furthermore, the life history parameters of cownose rays suggest they have low reproductive potential and their populations are incapable of rapid increases. Hypothesized trophic cascades should be closely scrutinized as spurious conclusions may negatively influence conservation and management decisions.

The concept and evidence for trophic cascades is well-documented from mesocosm studies and experiments conducted in simple terrestrial and marine systems^1^,^2^,^3^,^4^. In well-described examples from terrestrial ecosystems and simple food webs, the loss of top predators releases prey populations from predation mortality thereby resulting in inverse trends in abundances of predators and prey^5^. The effects may cascade through multiple trophic levels as increased abundance in the prey population results in higher than normal predation or overgrazing of their food source(s). However, the lack of empirical studies on marine trophic cascades, in part, stems from the challenge of acquiring quality long-term population data on predators and prey^6^,^7^,^8^,^9^,^10^.

There is an emerging concern with respect to the extensive use of non-linear phenomena such as regime shifts and trophic cascades to describe ecosystem changes without sufficient evidence to support these claims^2^,^8^,^9^,^10^. The lack of valid evidence is primarily attributed to the inadequacy of datasets with respect to temporal and spatial resolution and scope for evaluating change in relation to system variability, as well as insufficient demonstration of mechanistic links between pressures and ecological change^9^. Menge^3^ defined a trophic cascade as a tri-trophic interaction, typically where a predator indirectly influences primary production through direct influence on an herbivore. Primary producers are not theoretically necessary to establish a trophic cascade as long as a tri-trophic level interaction is observed^2^,^4^. Trophic cascades mediated by predation release should, at a minimum, require: 1) temporally correlated inverse abundance trends between predators and prey, 2) spatiotemporal overlap between predators and prey, 3) prey populations that grow rapidly compared to their predators, 4) specified prey must be a significant component of the predator’s diet and 5) specific predators included are the primary source of predation mortality on the prey.

Defining the role of large sharks in marine ecosystems has challenged ecologists for decades. Using terrestrial systems as models, it has been plausibly predicted that large sharks, as apex predators, may exert top-down influences on prey populations through direct predation or by triggering risk-averse behaviors that affect survivorship^11^. However, studies using empirical data with the Ecopath/Ecosim ecological modeling software^12^,^13^ or network analysis have suggested that sharks may not be strong regulators of community structure^14^,^15^,^16^ in large marine ecosystems. The relatively high diversity, functional redundancy, and complexity of marine food webs may attenuate potential cascading effects from the depletion of predators^17^.

Though evidence for trophic cascades in complex marine systems caused by the removal of sharks remains equivocal, Myers *et al.*^18^ proposed that significant declines in large coastal sharks in the northwest Atlantic Ocean caused dramatic increases in abundances of smaller elasmobranchs (referred to as “mesopredators”) through predation release. The large coastal shark decline purportedly led to an order of magnitude increase in the population of cownose rays, *Rhinoptera bonasus*, thereby increasing consumption of bivalve mollusks and leading to the collapse of commercial bivalve populations along the U.S. East Coast. This proposed trophic cascade has been widely cited and included as an important example of a marine trophic cascade in numerous book chapters and review papers^5^,^7^,^11^. This proposed trophic cascade, in addition to anecdotal fisher perceptions, was used to justify the development of a fishery for cownose rays as a form of predator control to decrease mortality rates on populations of commercially valuable bivalves. The cownose ray fishery was highly publicized through the “Save the Bay, Eat a Ray” campaign as a way to restore the health of Chesapeake Bay by facilitating recovery of collapsed filter-feeding oyster populations. Through the campaign, the fishery is being promoted by state fishery managers, seafood companies, some environmental organizations and local “eco-friendly” restaurants^19^,^20^,^21^.

The implications of this fishery have yet to be measured. There is concern that fishery management policies have been adversely influenced in unintended ways, which appears to be based on poor data interpretations, as well as a lack of consideration for the biology of the species in question or possible alternative explanations of observed patterns. In light of the recent discussions of trophic cascades^8^, we use the proposed shark-mediated tri-trophic cascade^18^ as a case study to critically examine the attribution and quality of evidence pertaining to all three trophic levels with respect to the five diagnostics listed above, introduce new analyses to help evaluate the case study claims and discuss the consequences of attributing the decline in bivalve populations to increased cownose ray consumption caused by predation release.

## Results and Discussion

A reexamination of the existing data, as well as incorporation of additional information, indicate that the marine trophic cascade proposed by Myers *et al.*^18^ is not well-supported. Though a compelling hypothesis, closer scrutiny of population patterns and trophic linkages (described in detail below) reveal a lack of correlation and mechanistic causation. At a minimum, alternative interpretations imply that trophic cascades triggered by the declines of apex predatory sharks in the ecosystem examined by Myers *et al.*^18^ are equivocal. Therefore, caution should be applied in any management policies invoking the results of that study. Five questions were addressed in detail and highlight the failure of this case study to meet the diagnostic criteria for a trophic cascade (Table 1).

### How reliable are the relative abundance trends for predators?

The magnitudes of predator declines are highly relevant and one should strive to use the most accurate and defensible estimates for these declines. In their proposed shark-mediated trophic cascade, Myers *et al.*^18^ reported an 87% decline in sandbar sharks and a 99% decline in dusky sharks based on a single dataset with limited geographic range. Prior to the Myers *et al.*^18^ publication, the available stock assessments^22^,^23^ (using many datasets, including the VIMS survey discussed later) for U.S. Atlantic sandbar (*Carcharhinus plumbeus*), and dusky sharks (*C. obscurus*), indicated a decline of 59% and 80%, respectively, during the same time frame. For sandbar sharks, if the decline was 59%, the remaining population is 320% (3.2 times) of the remaining population if the decline was 87%. For dusky sharks, there is a 2000% (20-fold) difference in the remaining population depending on whether the decline was 80% or 99%. This has tremendous ramifications for the potential for recovery of these populations under different management regimes.

Myers *et al.*^18^ used a fishery-independent longline survey conducted by the University of North Carolina (UNC) as the primary data source to estimate abundance trends in eight species of large coastal sharks implicated in the proposed shark-mediated trophic cascade. Additionally, fishery-independent trawl surveys and fishery-dependent pelagic longline data were examined for abundance trends, but these data sources do not adequately sample or target large coastal shark species^24^ and were not used in support of the trophic cascade. Based on the UNC indices, seven of eight species of large coastal sharks analyzed (including three species with sample sizes of only 5–39 individuals throughout the survey time series; refer to METHODS) showed declines between 87% and 99%^18^. The strength of the UNC survey lies in its longevity (1972 – present); however, it is extremely limited geographically and includes only two fixed nearshore stations off North Carolina. In spite of the spatial limitations, it was hypothesized that this survey reflected population-wide changes in abundance since the two stations lie in the migratory path of many highly migratory shark species. This hypothesis is easily testable as the Virginia Institute of Marine Science (VIMS) conducts a fishery-independent longline survey of similar longevity (1974 – present) that includes five fixed stations stratified by depth in Virginia coastal waters^25^. Both (UNC and VIMS) are fixed-station surveys designed to measure relative abundance of coastal shark populations along the U.S. East coast. The surveys are ~200 km apart and lie along the migratory path of all of the species involved in the purported trophic cascade, therefore, they should produce similar trends in relative abundance if they track stock-wide changes in abundance of these highly migratory species.

**Figure 1:**
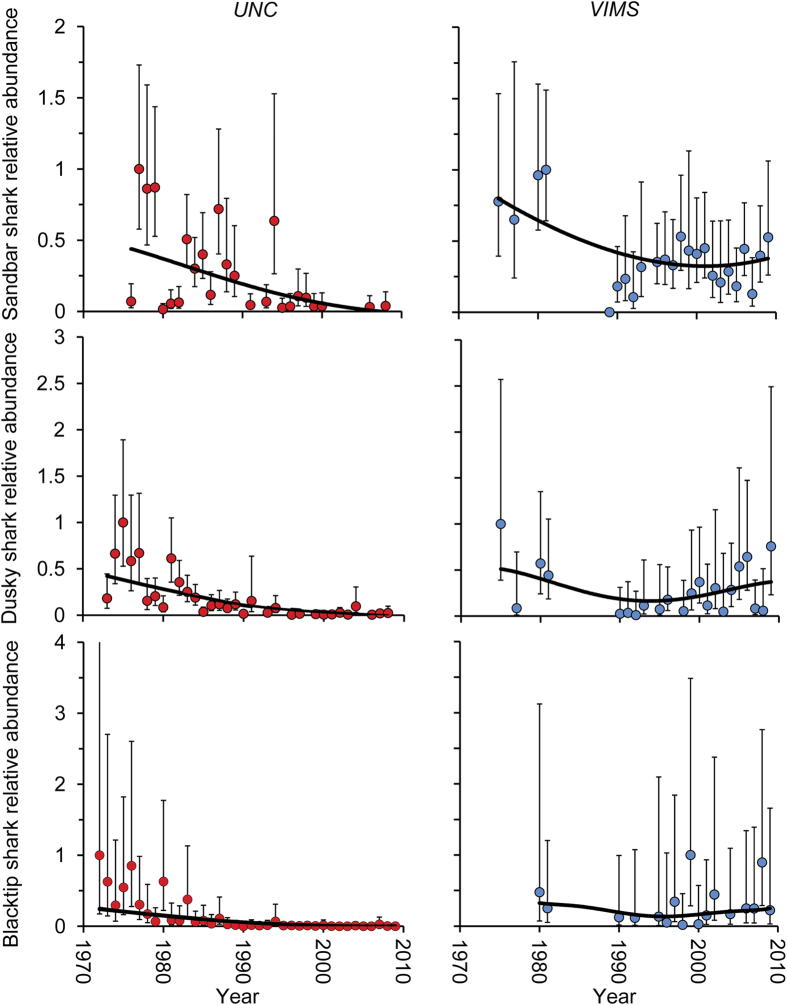
Estimated relative abundance for sandbar sharks, dusky sharks, and blacktip sharks from the University of North Carolina (UNC) and Virginia Institute of Marine Science (VIMS) shark longline surveys with 95% confidence intervals. Relative abundance is expressed as the year’s estimated mean index divided by the maximum estimated yearly mean index in each time series. Trend lines are estimated based on the delta-lognormal model fitted responses and plotted using loess smoothing.

Changes in relative abundances for all species varied greatly between the UNC and VIMS fishery-independent surveys (Fig. 1). The relative abundances of sandbar, dusky, and blacktip (*Carcharhinus limbatus*) sharks in the UNC survey declined dramatically over the survey period (1972–2009) to the point that all three species were uncommon after 1990. In contrast, the recent relative abundances of these three species in the VIMS survey are higher than they have been in at least 20 years. Sandbar and dusky sharks have historically been the most common large coastal shark species captured in the VIMS survey^25^. Relative abundances of both species declined dramatically throughout the 1980 s as the fishery for large coastal sharks developed and management regulations were lacking. Relative abundance of sandbar sharks increased through the late 1990 s and has been highly variable in recent years, fluctuating between 20% and 60% of peak levels. Dusky shark relative abundance in the VIMS survey has increased steadily since the early 1990 s and recent catch rates are comparable to historic highs. Blacktip sharks historically were not abundant in the VIMS survey, but the relative abundance of this species has increased steadily over the past 20 years and is currently at a historic high.

Whereas sharks declined in both the UNC and VIMS surveys during the 1980 s, the disparate trends beginning in the 1990 s refute the hypothesis that either survey reflects population-wide changes in abundance. Correlation coefficients between the UNC and VIMS survey using the model estimates (1990–2009) for sandbar, dusky and blacktip shark relative abundance are 0.093, −0.311, and −0.224, respectively, with no significant correlations (p > 0.05) (Fig. S1). Sharks did not show any signs of recovery in the UNC time series while relative abundance began to increase in the 1990 s in the VIMS survey coinciding with the first Federal Fishery Management Plan for Sharks and subsequent regulations that included trip limits and quotas^26^. If either of these surveys accurately reflects population-wide changes in abundance, it would be predicted that stock assessments might demonstrate similar trends. Recent stock assessments using indices from many data sources indicated the biomass in 2009 of sandbar sharks and dusky sharks in the northwest Atlantic Ocean and Gulf of Mexico had declined by 66% and 80%, respectively, relative to unfished conditions^27^,^28^. These declines are considerably less severe than the 87% decline for sandbar shark and 99% decline for dusky shark based only on the UNC survey but more severe than indicated by the VIMS survey. Results of the stock assessments indicate that sandbar shark biomass has stabilized since 2007 and although the dusky shark biomass continued to decline through 2009, the abundance (number of sharks) has been increasing since 2004 27,28, an expected sign of early recovery due to the time lag for juveniles to recruit to the adult population. The status of the blacktip shark stock is unknown in the Atlantic Ocean, but a stock assessment in 2011 for the Gulf of Mexico stock concluded the biomass in 2010 was at 87% of unfished conditions and is not overfished^29^.

The VIMS survey shows more optimistic trends for all of these species. This may be partially because the VIMS survey is located within known shark nursery habitat for sandbar and dusky sharks^23^. However, given the close proximity and similar methods employed by the VIMS and UNC surveys analyzed here, the disparate trends in relative abundance for all three species could be explained by a slight northern shift in the seasonal migration patterns of these species. Considering all data sources, we conclude that large coastal shark populations did decline during the 1970 s and 1980 s, but not as dramatically as described^18^ and these populations have at least started to recover. In addition, several other shark populations have been recovering since the 1990 s^30^,^31^ when the U.S. initiated management of federal shark fisheries. These results illustrate the dangers of broadly interpreting results from a single index with limited scope and applying those results to an entire population.

### Is there spatiotemporal overlap between predators and mesopredators?

**Figure 2:**
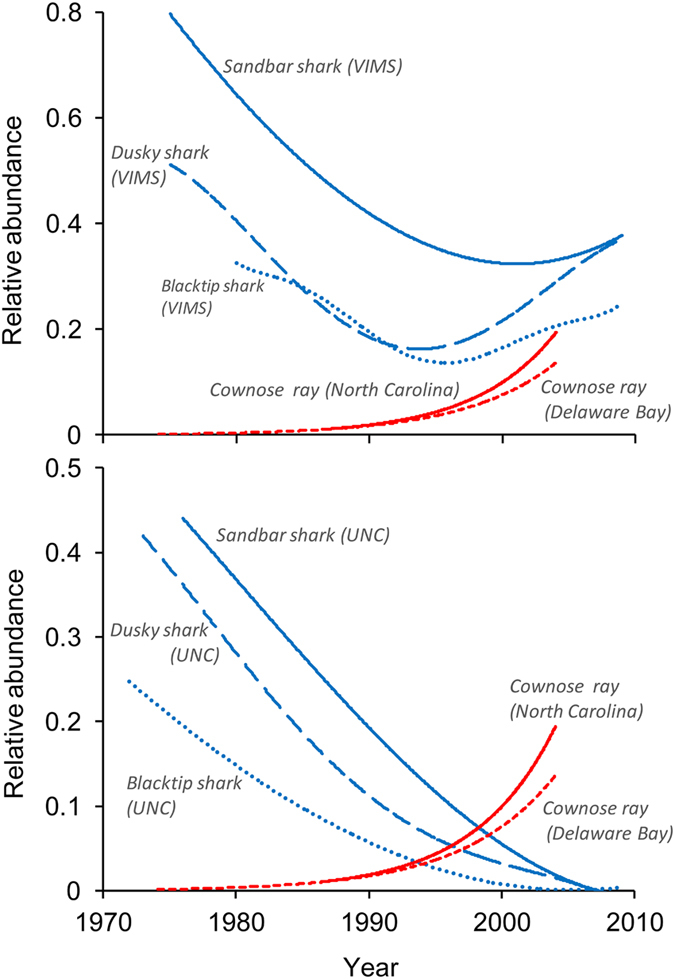
Trends in estimated relative abundance for sandbar sharks, dusky sharks, and blacktip sharks from the Virginia Institute of Marine Science (VIMS) and the University of North Carolina (UNC) longline surveys compared to trends in cownose ray relative abundance in the Delaware Bay, Delaware and Pamlico Sound, North Carolina trawl surveys. Data on cownose ray abundance are from Heithaus *et al.*^11^. Relative abundance is expressed as the year’s estimated mean index divided by the maximum estimated yearly mean index in each time series.

Our findings show a lack of temporal correlation between predator (large coastal sharks) and consumer (cownose ray) abundance, and therefore, provide no support for a predation-mediated trophic cascade. The cownose ray is the primary “mesopredator” (consumer) directly implicated by Myers *et al.*^18^ in the reduction of bivalve populations. Cownose ray relative abundance was reported to increase in six of the seven survey indices examined and hypothesized that the increase was a result of predation release due to declines in large sharks. Data documenting the increase in the cownose ray population came largely from surveys in the Mid-Atlantic Bight region. Whereas spatial overlap occurs between cownose rays and large sharks in this region, temporal abundance trends of predators and purported prey do not support the trophic cascade hypothesis. A comparison of data from the two surveys that documented the most drastic increases in cownose rays (the Delaware Department of Natural Resources and Environmental Control, DNREC, trawl survey conducted in Delaware Bay, Delaware and the North Carolina Division of Marine Fisheries, NCDMF, trawl survey conducted in Pamlico Sound, North Carolina)^11^ and the data from the VIMS shark survey indicate that the temporal increase in cownose rays coincided with an increase (not a decrease) in sandbar, dusky, and blacktip sharks based on estimated trends in abundance (Fig. 2). Correlations between cownose ray (from both Delaware Bay and North Carolina) and VIMS shark relative abundance from 1990 to 2004 were primarily positive; whereas, the majority of correlation coefficients for comparisons between cownose ray and UNC shark relative abundance during the same time frame were negative (Fig. S1). Support for the hypothesis that an increase in the cownose ray population resulted from predation release due to declines in predator abundance depends on the survey chosen (UNC or VIMS) and is therefore equivocal. Only one of the correlations between cownose rays and sharks for all surveys combined had significant results (α = 0.05; the positive correlation between Delaware Bay cownose ray and VIMS sandbar shark abundance, Fig. S1). There is also a lack of support in general for population increases via predation release for other mesopredators in the region such as Atlantic sharpnose shark (*Rhizoprionodon terraenovae*) and blacknose shark (*Carcharhinus acronotus*). Stock biomass trajectories for blacknose shark declined steeply from about 1988 to 1994, followed by a precipitous decline from 1994 to 1995 in the northwest Atlantic Ocean and Gulf of Mexico^32^. Similarly, the abundance of Atlantic sharpnose sharks was about 50% of unfished condition in the early 1990 s^33^. These declines coincided with an overall decrease in larger predatory sharks, not an increase as the “mesopredator release hypothesis” predicts.

### Are mesopredator population growth rates realistic?

**Figure 3:**
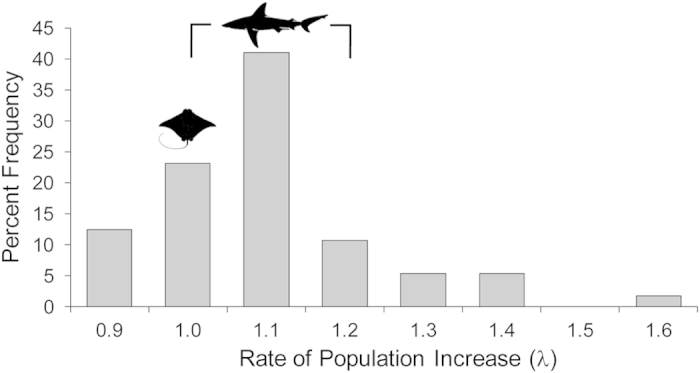
The percent frequency of the population growth rates (λ) for 38 species of sharks^34^ and 5 species of skates^35^ as a comparison to that of the cownose ray and the “great sharks” as considered by Myers *et al.*^18^. The finite rate of population increase (λ) was calculated assuming *r* is analogous to the intrinsic rate of population increase (e^*r*^ = λ). The images of the cownose ray and shark are available at http://www.nefsc.noaa.gov/lineart.

Trophic cascades mediated by predation require that prey populations grow rapidly compared to their predators^2^. However, based on our demographic analyses, cownose rays exhibit lower population growth rates than many of the proposed top level predators. Myers *et al.*^18^ reported cownose ray populations increased at a mean meta-analytic population growth rate (*r*) of 0.087 yr^−1^. Such growth was possible due to assumptions of no fishing mortality and natural mortality less than 0.076 yr^−1^ 18. This value of natural mortality does not seem plausible given the range of values estimated for other elasmobranchs^34^,^35^ and such high population growth rates are inconsistent with the biology of the species. Current life history data^36^ indicate that cownose rays are late maturing (7–8 years) and have only one offspring per year, yielding one of the lowest lifetime fecundity estimates (<14) of any fish species and are therefore incapable of rapid population increases. Using these parameters as inputs to a traditional life table (see METHODS, Cownose ray productivity), we estimated that population growth rates (*r*) are nearly stable, ranging from −0.018 yr^−1^ to 0.032 yr^−1^ (median *r* = 0.008). Cownose rays are indeed among the least productive elasmobranchs (Fig. 3). The high rates of population increase reported in the proposed cascade^18^ are better explained by high model uncertainty, sampling bias with the surveys (e.g., changes in survey design), or distribution shifts in the cownose ray population. Survey bias and changes in distribution have been identified as issues to consider in monitoring elasmobranch abundance trends in the northwest Atlantic^37^, and also likely influence cownose ray detection rates in the available surveys. These issues are compounded for species like cownose rays that travel in very large schools. Given the updated life history data and demographic analysis, the cownose ray does not exhibit rapid population growth in comparison to the large coastal sharks purported to exhibit top-down control of these consumers (Fig. 3).

### How strong are predator-mesopredator trophic linkages?

**Figure 4:**
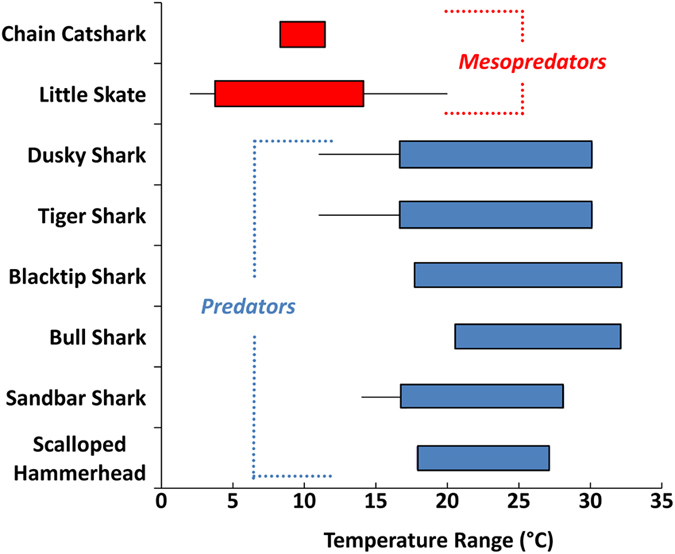
Thermal tolerances indicate a lack of spatiotemporal overlap between large coastal sharks and some of their purported “mesopredator” prey^18^. Boxes represent normal temperature ranges and horizontal lines represent maximum and minimum tolerances (where such data were available). Data were sourced from the literature^72^,^73^,^74^,^75^,^76^ and the VIMS longline database.

There is little doubt that large coastal sharks worldwide feed on smaller sharks and rays. However, there is no evidence to indicate that cownose rays or other elasmobranch mesopredators are a significant component of the diet for large coastal sharks, therefore, weakening the evidence for this trophic link in the purported trophic cascade. Myers *et al.*^18^ examined abundance trends of 14 species of mesopredators, but most showed insignificant, equivocal or declining trends. Five species showed significant increases that were reported to result from trophic release due to large shark declines. Due to different thermal tolerances, some of these, such as the chain catshark (*Scyliorhinus retifer*) and the little skate (*Leucoraja erinacea*), lack the spatiotemporal overlap with the large sharks to allow trophic release (Fig. 4) and temporal disparity in relative abundance of some small coastal sharks was discussed above. Cownose rays were the primary mesopredator implicated in the tri-trophic cascade and cownose rays are not a significant component of the diet of any large coastal shark. As a whole, chondrichthyans make up less than 13% of the diet of all but two species (great hammerhead (*Sphyrna mokarran*) and bull sharks (*Carcharhinus leuca*s)) of large sharks considered within the proposed trophic cascade^18^. Unfortunately, elasmobranchs in the stomachs of large sharks are often not identified beyond the family level, making speculation about the species tentative at best. Upon review of 39 published diet studies for the large coastal shark species considered^18^, we determined that cownose rays have been identified only in the stomachs of blacktip and sandbar sharks in the northwest Atlantic, but at low frequencies of occurrence (3%, n = 6 of 174 sharks; 0.3%, n = 2 of 608 sharks; respectively)^38^,^39^. Most studies on the diets of large coastal sharks from the Mid-Atlantic region indicate skates (Rajidae) rather than cownose rays are the primary elasmobranch in the diet. Generic skates contributed 8.5% of the diet of dusky shark and little skate (*Leucoraja erinacea*) made up 5.9% of the sandbar shark diet in the northern part of their range^40^,^41^. Spiny dogfish (*Squalus acanthias*), dusky smoothhounds (*Mustelus canis*), dasyatid rays, and carcharhinid sharks all occurred more often than cownose rays in shark diets in the U.S. Mid-Atlantic region^39^,^40^,^41^.

Species other than large sharks also prey on cownose rays though it was asserted that large sharks are the only significant source of predation mortality^18^. For example, cownose rays were found in 9% of cobia (*Rachycentron canadum*) stomachs in Chesapeake Bay, VA^42^, a higher proportion than published diet studies for any shark species in the northwest Atlantic. Overall, there is little evidence to suggest large coastal sharks in the Mid-Atlantic region are sufficient sources of natural mortality on cownose rays to exert significant top-down regulation of their populations.

### Does the mesopredator negatively affect consumer/resource populations?

**Figure 5:**
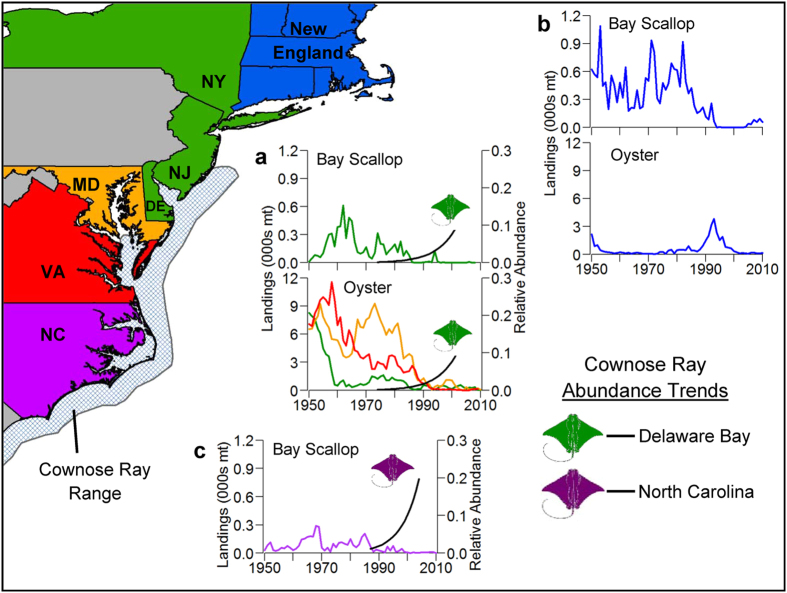
ArcGIS map of the U.S. Atlantic coast and plots of relative abundances of cownose rays (black lines) in the Delaware Bay, DE and Pamlico Sound, NC trawl surveys compared to landings data (colored lines) for bay scallop and eastern oyster from the Mid-Atlantic and northeast U.S. Atlantic coast for all available years (National Marine Fisheries Service landings). Line colors match the colors of the corresponding state/region on the map. The cross-hatched area delineates the core range of cownose rays in the region. Data on cownose ray abundance are from Heithaus *et al.*^11^.

A further hypothesized consequence of the tri-trophic cascade is cownose rays have driven commercial bivalve populations along the U.S. East Coast to collapse^18^. However, it is implausible that cownose rays were responsible for the observed declines in commercial bivalve landings. Timing of the purported increase in the cownose ray population in the 1990 s does not overlap with the well-documented and long-term declines in those commercial bivalve stocks (Fig. 5). This temporal disconnect is especially pronounced in Chesapeake Bay where oyster (*Crassostrea virginica*) landings in Virginia and Maryland were at all-time lows by the middle of the 1980 s. Following the historic declines in Virginia and Maryland oyster landings, there was a slightly negative correlation between these landings and Delaware Bay cownose ray abundance from 1980 through 2004 (Fig. S2). This correlation was not significant, even given time lags of up to 5 years to account for growth to recruitment age for oyster fisheries in that region (Fig. S2). Maryland oyster landings did have a significant negative correlation with North Carolina cownose ray abundance from 1980 to 2004 (Fig. S2). The spatial overlap of these two indexed populations is likely limited. The North Carolina trawl survey used to produce the cownose ray abundance estimates is conducted in June and September and the majority of cownose rays in Maryland waters are present from May throughout the summer and begin their migrations south during the month of September^18^,^36^. Bay scallop (*Argopecten irradians*) landings in the Mid-Atlantic states follow similar patterns as seen in the oyster landings, with collapses by the middle of the 1980 s (Fig. 5a). Large increases in the abundance indices of cownose rays did not occur until the early to middle 1990 s. Following the historic declines, correlation analysis only showed a significant negative correlation of Mid-Atlantic bay scallops with cownose rays from the region (Delaware Bay) when using a 3-year lag in scallop landings (Fig. S2) and by age 1, bay scallops reach sexual maturity, spawn, and are recruited to the fishery^43^. Since the onset of bay scallop and oyster negative trends from the Mid-Atlantic were not concurrent with increases in cownose ray abundance, it is impossible that cownose rays were the cause of long-term bivalve declines in this region. Furthermore, oyster and bay scallop populations have declined in areas outside the geographical range of cownose rays, e.g. New England^44^,^45^ (Fig. 5b). The decline in oyster populations along the U.S. East Coast primarily resulted from overharvest^44^,^45^,^46^ with recovery hindered by pollution and shellfish diseases such as MSX (*Haplosporidium nelsoni*) and dermo (*Perkinsus marinus*)^47^.

To support the hypothesis that a tri-trophic cascade caused cownose rays to drive commercial bivalve populations to collapse, Myers *et al.*^18^ cited experiments conducted in 1996 and 1998 on Oscar Shoal, North Carolina and found predator exclusion stockades reduced late season cownose ray predation on scallops^48^. However, there was no conclusive evidence that the stockades did not also exclude other predators. Gulls are mentioned as a potential predator causing late season (fall) declines in North Carolina scallops in an earlier paper by the same author that conducted the stockade experiments^49^. These experiments were not repeatable at other sites at that time, and the severe declines at Oscar Shoal were not found to occur at other nearby sites with substantial scallop populations^50^. This study also reported that years of low early summer density lacked the late summer site extinction and suggested that cownose rays feed elsewhere when populations are low. The authors concluded that the extinction at the Oscar Shoal site is density dependent (the scallop populations were the highest in the region) and site-specific due to the hydrography. This study supports the notion that predators commence feeding in high density prey patches, and that models should be spatially explicit. However, the extrapolation of these studies to a region wide predatory decline is unrealistic, given the limited temporal span of the stockade experiments and the lack of consideration for other factors that may exhibit pressure on these resources, such as habitat differences and additional sources of natural mortality^10^. Widespread bay scallop declines have been attributed to overharvest, habitat degradation, and harmful algal blooms^43^,^50^,^51^,^52^, all of which have contributed to recruitment failures. For example, the well-documented collapse in North Carolina’s bay scallop population (see Fig. 5c) was due to an acute, catastrophic red tide that occurred in 1987–1988 52,53,54, before the increase in cownose ray abundance was evident in the early to mid 1990 s. Correlation analyses of North Carolina cownose ray abundance and North Carolina bay scallop landings, including lags in scallop landings for up to 5 years, show no correlations between the two time series (Fig. S2). Due to their biology, recruitment limits scallop population size and low densities result in low recruitment^55^, which limits their recovery from depleted populations. The most any predators could contribute to scallop population dynamics is to limit recovery of a collapsed population. Acknowledging that bivalve landings, as used here, may represent poor estimates of stock biomass, it is clear that the historic declines in these commercial bivalve stocks do not temporally correlate with increases in cownose ray abundance.

There is little quantitative support for the notion that cownose ray predation drives commercial bivalve population trends. Based on published diet studies of cownose rays, predation on wild hard-shelled commercial bivalves such as oysters and hard clams is minimal as these were identified in less than 3% of stomachs examined in Chesapeake Bay^56^. Cownose rays are capable of consuming weaker shelled commercial bivalves such as soft-shelled clams^56^ but small non-commercial bivalves, crustaceans, polychaetes, and echinoderms are more common components of the cownose ray diet^56^,^57^,^58^,^59^. The “Save the Bay, Eat a Ray” campaign stemmed from anecdotal reports of intense predation on small cultchless oysters in commercial aquafarms. Contradicting these reports, oysters constituted only 5% of the diet of cownose rays harvested on commercial oyster grounds in Chesapeake Bay^59^. Furthermore, gape and bite force limitations may restrict the ability of cownose rays to eat large oysters that are typically present in these areas^60^. The structural complexity of wild, cultched oysters on natural reefs also mechanically limits access for predation^59^.

## Conclusions

Collectively, our new analyses indicate there is little support for the hypothesis of a shark-mediated trophic cascade in the coastal Mid-Atlantic system. Critical review of the evidence provided for the purported trophic cascade with respect to five diagnostics for each trophic linkage indicated that only one criterion was met for the large coastal shark – cownose ray link and two criteria were met for the cownose ray – bivalve link (Table 2). Spatiotemporal overlap was demonstrated for both linkages; although, the cownose ray – bivalve link is seasonal and is spatially limited. Additionally, the criterion for prey population growth to be rapid compared to the predator was met for the cownose ray – bivalve link (Table 2). When considering additional data sets and robust stock assessments, large shark population declines were not as severe as reported and did not coincide with reported increases in the abundances of smaller elasmobranchs, particularly cownose rays. Similarly, evidence is lacking to support the conclusion that higher cownose ray abundance led to the collapse of commercial bivalve populations as bivalve stocks had undergone dramatic declines due to other causes more than a decade before the reported increases in cownose rays occurred. Yet, a fishery for cownose rays was promoted under the guise of predation control in the hopes of rebuilding natural oyster populations and promoting a growing bivalve aquaculture industry. Cownose rays are natural predators in these systems and may inhibit some efforts to mitigate for shellfish stock declines that resulted from overfishing, disease, and habitat loss, but there is no evidence that cownose rays were the cause of the declines, but rather have been portrayed as scapegoats for these declines.

The consequences of the frequently cited trophic cascade in the northwest Atlantic Ocean^18^ and the subsequent development of the unregulated “Save the Bay, Eat a Ray” fishery may be detrimental to this population of slow-growing elasmobranchs with limited intrinsic growth rate and rebound potential. Based on the life history of the cownose ray, it is unlikely the species can withstand high levels of fishing mortality without substantial population declines. Recent reported annual landings of cownose rays in Chesapeake Bay have been as high as 186 metric tons. By comparison, the current U.S. federal quota for “Aggregated Large Coastal Sharks” in the Atlantic is 168.9 metric tons. There are no estimates of cownose ray population size and have been no efforts to estimate what level of fishing mortality these landings represent. In addition, these estimates do not take into account landings in other regions along their migratory path or unreported mortality through recreational bow-fishing tournaments and bycatch discards from other fisheries. Establishment of precautionary, science-based limits rather than continuation of unregulated cownose ray fisheries will limit the potential for population collapse.

Our analysis underlines the potential consequences of misinterpreting results from a single survey index with limited scope, and the importance of closely analyzing data on diet, predator-prey co-occurrence and temporal correlation of population trends before inferring trophic cascades with meta-analyses. While large coastal sharks have important ecological roles as upper level marine predators, we conclude that their population declines have not been significant enough to elicit trophic cascades at the scale hypothesized by Myers *et al.*^18^. Spurious conclusions from such studies may lead to counterproductive conservation and management policies. As such, close scrutiny of alleged trophic cascades is required, particularly when alternative explanations of population trends are not explored.

## Methods

### Abundance trend analysis

We re-analyzed the University of North Carolina (UNC) and Virginia Institute of Marine Science (VIMS) data sets through 2009 for three large coastal shark species, sandbar shark, dusky shark, and blacktip shark, using the Delta method^61^. These are the most abundant large coastal shark species shared by both surveys. Myers *et al.*^18^ suggested large population declines (up to 99%) occurred in four additional large coastal sharks; scalloped hammerhead (*Sphyrna lewini*), smooth hammerhead (*S. zygaena*), bull shark (*Carcharhinus leucas*) and tiger shark (*Galeocerdo cuvier*). Three of these species occur too infrequently in either survey to produce credible population trends. Over the 32-year span of the UNC survey through 2005, only 5 smooth hammerhead sharks, 23 bull sharks, and 39 tiger sharks were captured, yet declines of 97–99% were reported by Myers *et al.*^18^. Due to low sample sizes we did not provide any analysis, as any comparison would be inappropriate. Juvenile scalloped hammerheads, historically common in the UNC survey, were encountered too infrequently in the VIMS survey to allow comparison.

Catch per unit effort (CPUE) in number of sharks per hook was used to examine the relative abundance of sharks caught during all surveys. The relative abundance indices were estimated by fitting two ‘sub-models’ to the data^61^. The first sub-model involves modeling the probability of a non-zero catch, assuming a binomial error distribution and a logit link function, and a second sub-model where the catch is nonzero and the distribution is assumed to be lognormal. The delta-lognormal modeling approach has been described extensively elsewhere^62^, and is the primary method used for modeling fishery-independent survey data used in stock assessments during the Southeast Data Assessment and Review process for U.S. Atlantic shark stocks^27^,^28^,^29^.

For the UNC data, the factors considered as potential influences on the CPUE for these analyses were: year (1972–2009), month (April – November), station (E-W, N-S), and temperature (<20 °C, 20–24 °C, 25–29 °C, and > 30 °C). For the VIMS data series, the factors included were: year (1975–2009), month (June–September) and station (five fixed stations). Factors most likely to influence abundance were evaluated in a forward stepwise fashion^63^. Initially, a null model was fit, then single factor models were fit to the data. Each single factor model was ranked from greatest to least reduction in deviance per degree of freedom when compared to the null model. The factor associated with the model that resulted in the greatest reduction in deviance was then incorporated into the model providing the effect was significant at p < 0.05 based on a chi-squared test, and the deviance per degree of freedom was reduced by at least 1% from the less complex model. The process was continued until no factors met the criterion for incorporation into the final model. Regardless of its level of significance, year was retained in all models. This allows the estimation of the annual indices, which was the main objective of the standardization process, but also accounted for the variability associated with year interactions^64^. After selecting the set of factors for each error distribution, all interactions that included the factor year were treated as random interactions^64^. The standardized CPUE values for the Delta models were calculated as the product of the expected probability of a non-zero catch and the expected conditional catch rate for sets that had a non- zero catch. The expected probability and expected conditional catch rate were the least square means of the factor year from each of the two analyses that constitute an analysis using the Delta model approach^61^,^62^,^63^,^64^,^65^. All models were fit using a SAS macro, GLIMMIX (glmm800MaOB.sas: Russ Wolfinger, SAS Institute Inc.) and the MIXED procedure in SAS statistical computer software (PROC GLIMMIX). Final models were selected based on Akaike Information Criteria (AIC).

### Cownose ray productivity

To evaluate the proposed rate of population increase, we constructed deterministic age-structured life tables for female cownose rays to estimate population growth rates following established methods for elasmobranchs^66^,^67^. Uncertainty in life history parameters (Table 3) was considered using probabilistic distributions by varying age-specific vital rates using Monte Carlo simulation^67^. This approach involves random, independent resampling of life history parameters with replacement from theoretical probability density functions. The instantaneous rate of population increase (*r*) was iteratively solved using the discrete form of the Euler–Lotka equation:


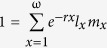


where *l*_*x*_ is the probability of an individual being alive at the beginning of age *x*, *m*_*x*_ is the number of offspring produced annually by individuals at age *x* and *ω* is the maximum age. Initial values of *l*_*x*_ (age 0) were set at 1.0. The range and mean of the instantaneous rate of population increase were calculated and 95% confidence intervals were calculated as the values bounded by the 97.5th upper and 2.5th lower percentiles.

It was previously assumed that cownose ray would have a constant mortality throughout their life span^18^, which seems implausible for a species that is born at approximately 400 mm disk width and reaches a maximum size of about 1200 mm disk width^36^. Life history theory would predict that mortality initially decreases with age, but remains fairly stable once individuals attain a certain size or age and predation risk decreases. It has been argued for elasmobranchs, methods providing age-specific estimates of mortality are more biologically sound than those providing a single value for all ages^67^. Following that argument, the instantaneous rate of natural mortality (converted to survivorship) was estimated through multiple indirect life-history methods to incorporate the range of mortality mechanisms (e.g. temperature-dependent, age-scheduling). One method relies on estimates of longevity^68^, whereas another uses parameters estimated through the von Bertalanffy growth model and water temperature^69^. The age-specific method^70^ estimates natural mortality (*M*) based on body mass-at-age and the final method incorporates two different equations, one to describe mortality of the early life stages and the second one for older ages^71^. This function generates high initial mortality estimates that decrease with size and increase again during senescence. For species that are undergoing exploitation, the maximum age-specific estimate of survival from the multiple invariant methods was used to simulate a density-dependent response^69^. Variability in the estimates of survivorship was determined from the standard deviation of all methods used.

## Additional Information

**How to cite this article**: Grubbs, R. D. *et al.* Critical assessment and ramifications of a purported marine trophic cascade. *Sci. Rep.* **6**, 20970; doi: 10.1038/srep20970 (2016).

## Supplementary Material

Supplemental Figures 1 and 2

## Figures and Tables

**Table 1:** Critical examination of the case study based on diagnostic criteria for a trophic cascade.

Diagnostic	Question
Temporal correlation of abundance trends	How reliable are the relative abundance trends for predators?
Spatiotemporal overlap	Is there spatiotemporal overlap between predators and mesopredators?
Prey growth rapid compared to predator	Are mesopredator population growth rates realistic?
Prey significant part of predator diet	How strong are predator-mesopredator trophic linkages?
Predator primary source of predation mortality	Does the mesopredator negatively affect consumer/resource populations?

**Table 2:** Results from the evaluation of the proposed large-scale marine trophic cascade between large coastal sharks, cownose ray, and bivalves using five diagnostic criteria for each trophic linkage.

Trophic linkage	Temporal correlation of abundance trends	Spatiotemporal overlap	Prey population growth is rapid compared to predator	Prey significant part of predator diet	Predator primary source of predation mortality
Large coastal sharks – cownose ray (predator – consumer)	NO	YES	NO	NO	NO
Cownose ray – bivalves (consumer – resource)	NO	YES	YES	NO	NO

**Table 3:** Life history and baseline parameters^33^ used to develop the cownose ray life table.

Parameter	Value	Standard deviation	Range	Distribution
Age-at-maturity:	a = −12.473			
	b = 1.945			
Maximum age:	21 years	4.04		Lognormal
Litter size:	1		0.9–1.1	Uniform
Reproductive periodicity	Annual			
Sex ratio	1:1			
Theoretical maximum size (L_∞_)	105.34 cm disc width	0.76		
Growth coefficient (*k*)	0.1931 yr^−1^	0.008		
Theoretical time at zero length (t_0_)	−2.64 yr			
Survivorship:				
Age 0–4	0.83 yr^−1^		0.62–0.83	Uniform
Age 5	0.84 yr^−1^		0.78–0.84	Uniform
Age 6–8	0.85 yr^−1^		0.79–0.85	Uniform
Age 9–15	0.86 yr^−1^		0.80–0.86	Uniform
Age 16–21	0.87 yr^−1^		0.82–0.87	Uniform

Distribution is the assumed distribution of the probability density function used in the Monte Carlo simulation.

## References

[b1] Paine R. T. Food webs: Linkage, interaction strength and community infrastructure. J. Anim. Ecol. 49, 667–685 (1980).

[b2] Polis G. A., Sears A. L. W., Huxel G. R., Strong D. R. & Maron J. When is a trophic cascade a trophic cascade? Trends Ecol. Evol. 15, 473–475 (2000). 11050351 10.1016/s0169-5347(00)01971-6

[b3] Menge B. A. Indirect effects in marine rocky intertidal interaction webs: patterns and importance. Ecological Monographs 65, 21–74 (1995).

[b4] Pinnegar J. K. *et al.* Trophic cascades in benthic marine ecosystems: lessons for fisheries and protected-area management. Environmental Conservation 27, 179–200 (2000).

[b5] Terborgh J. A. & Estes J. A. Eds. Trophic Cascades: Predators, Prey, and the Changing Dynamics of Nature (Island Press, Washington DC, 2010).

[b6] Shears N. T. & Babcock R. C. Continuing trophic cascade effects after 25 years of no-take marine reserve protection. Mar. Ecol. Prog. Ser. 246, 1–16 (2003).

[b7] Baum J. K. & Worm B. Cascading top-down effects of changing oceanic predator abundances. J. Anim. Ecol. 78, 699–714 (2009). 19298616 10.1111/j.1365-2656.2009.01531.x

[b8] Krebs C. *On tipping points and regime shifts in ecosystems.* (2015) Available at: https://www.zoology.ubc.ca/~krebs/ecological_rants/? p=1226 (Accessed: 5th May 2015).

[b9] Capon S. J. *et al.* Regime shifts, thresholds and multiple stable states in freshwater ecosystems; a critical appraisal of the evidence. Science of the Total Environment 517, in press 10.1016/j.scitotenv.2015.02.045. (2015). 25712747

[b10] Mac Nally R., Albano C. & Fleishman E. A scrutiny of the evidence for pressure-induced state shifts in estuarine and nearshore ecosystems. Austral Ecology 39, 898–906 (2014).

[b11] Heithaus M. R., Frid A., Wirsing A. J. & Worm B. Predicting ecological consequences of marine top predator declines. Trends Ecol. Evol. 23, 202–210 (2008). 18308421 10.1016/j.tree.2008.01.003

[b12] Christensen V. & Pauly D. ECOPATH II - a system for balancing steady-state ecosystem models and calculating network characteristics. Ecol. Model. 61, 169–185 (1992).

[b13] Walters C., Christensen V. & Pauly D. Structuring dynamic models of exploited ecosystems from trophic mass-balance assessments. Rev. Fish Biol. Fish. 7, 139–172 (1997).

[b14] Kitchell J. F., Essington T. E., Boggs C., Schindler D. E. & Walters C. J. The role of sharks and longline fisheries in a pelagic ecosystem of the central Pacific. Ecosystems 5, 202–216 (2002).

[b15] Carlson J. K. Modeling the role of sharks in the trophic dynamics of Apalachicola Bay, Florida, Amer. Fish. Soc. Symp. 50, 281–300 (2007).

[b16] Navia A. F., Cortés E. & Mejıa-Falla P. A. Topological analysis of the ecological importance of elasmobranch fishes: a food web study on the Gulf of Tortugas. Colombia. Ecol. Model. 221, 2918–2926 (2010).

[b17] Strong D. R. Are trophic cascades all wet? Differentiation and donor-control in speciose ecosystems. Ecology 73, 747–754 (1992).

[b18] Myers R. A., Baum J. K., Shepherd T. D., Powers S. P. & Peterson C. H. Cascading effects of the loss of apex predatory sharks from a coastal ocean. Science 315, 1846–1850 (2007). 17395829 10.1126/science.1138657

[b19] YouTube C. N. N. Channel. Eating rays may save Chesapeake Bay. (2010) Available at: https://www.youtube.com/watch?v=PYkA3KjZ1sU. (Accessed: 25 March 2015).

[b20] Virginia Seafood. Chesapeake Bay ray stories. (2004) Available at: http://www.virginiaseafood.org/chesray/stories.htm. (Accessed: 25 March 2015)

[b21] The Suburban Farm. Chesapeake (cownose) ray. (2010) Available at: http://www.suburbanfarm.com/2010/09/chesapeake-cownose-ray.html. (Accessed: 25 March 2015).

[b22] NMFS Southeast Data, Assessment, and Review SEDAR 11 Stock Assessment Report Large Coastal Shark Complex, Blacktip and Sandbar Shark. National Marine Fisheries Service, Highly Migratory Species Management Division, Silver Spring, MD. (2006) Available at: http://sedarweb.org/docs/sar/Final_LCS_SAR.pdf (Accessed: 17 September 2015).

[b23] Cortés, E., Brooks, E., Apostolaki, P. & Brown, C.A. Stock Assessment of Dusky Shark in the U.S. Atlantic and Gulf of Mexico.(2006) National Marine Fisheries Service, Highly Migratory Species Management Division, Silver Spring, MD. Sustainable Fisheries Division Contribution SFD-2006-014. Available at: http://www.nmfs.noaa.gov/sfa/hms/species/sharks/documents/2006_dusky_shark_assessment_for_distribution.pdf (Accessed: 17 September 2015).

[b24] Burgess G. H. *et al.* Is the collapse of shark populations in the northwest Atlantic Ocean and Gulf of Mexico real? Fisheries 30, 20–26 (2005).

[b25] Musick J. A., Branstetter S. & Colvocoresses J. A. “Trends in shark abundance from 1974 to 1991 for the Chesapeake Bight region of the U.S. Mid-Atlantic coast” (NOAA Technical Report NMFS 115 Conservation Biology of Elasmobranchs, Seattle, WA) (1993).

[b26] NMFS Fishery management plan for sharks of the Atlantic Ocean. National Marine Fisheries Service, Highly Migratory Species Management Division, Silver Spring, MD. (1993) Available at: http://www.nmfs.noaa.gov/sfa/hms/documents/fmp/shk_fmp/shk_fmp_1993.pdf (Accessed: 25 March 2015).

[b27] NMFS Southeast Data, Assessment, and Review SEDAR 21 HMS Dusky Shark SECTION V: Review Workshop Report. National Marine Fisheries Service, Highly Migratory Species Management Division, Silver Spring, MD. (2011) Available at: http://sedarweb.org/docs/sar/Dusky_SAR.pdf (Accessed: 25 March 2015).

[b28] NMFS Southeast Data, Assessment, and Review SEDAR 21 HMS Sandbar Shark SECTION V: Review Workshop Report. National Marine Fisheries Service, Highly Migratory Species Management Division, Silver Spring, MD. (2011) Available at: (http://sedarweb.org/docs/sar/Sandbar_SAR.pdf (Accessed: 25 March 2015).

[b29] NMFS Southeast Data, Assessment, and Review SEDAR 29 HMS Gulf of Mexico Blacktip Shark SECTION II: Assessment Report. National Marine Fisheries Service, Highly Migratory Species Management Division, Silver Spring, MD. (2012) Available at: http://sedarweb.org/docs/sar/S29_GOM%20blacktip%20report_SAR_final.pdf (Accessed: 25 March 2015).

[b30] Carlson J. K., Hale L. F., Morgan A. & Burgess G. R. Relative abundance and size of coastal sharks derived from commercial shark longline catch and effort data. J Fish Biol. 80, 1749–1764 (2012). 22497406 10.1111/j.1095-8649.2011.03193.x

[b31] Curtis T. H., McCandless C. T., Carlson J. K., Skomal G. B., Kohler N. E., Natanson L. J., Burgess G. H., Hoey J. J. & Pratt H. L. Jr. Seasonal distribution and historic trends in abundance of white sharks, *Carcharodon carcharias*, in the western North Atlantic Ocean. PLoS ONE 9(6), e99240 (2014). 24918579 10.1371/journal.pone.0099240 PMC4053410

[b32] NMFS Southeast Data, Assessment, and Review SEDAR 21 HMS Atlantic Blacknose Shark SECTION V: Review Workshop Report. (National Marine Fisheries Service, Highly Migratory Species Management Division, Silver Spring, MD. (2011) Available at: http://sedarweb.org/docs/sar/Atl_Blacknose_SAR.pdf. (Accessed: 25 March 2015)

[b33] NMFS Southeast Data, Assessment, and Review SEDAR 34 HMS Atlantic Sharpnose Shark SECTION V: Review Workshop Report. National Marine Fisheries Service, Highly Migratory Species Management Division, Silver Spring, MD, (2011) Available at: http://sedarweb.org/docs/sar/S34_ATSH_SAR.pdf. (Accessed: 25 March 2015).

[b34] Cortés E. Incorporating uncertainty into demographic modeling: application to shark populations and their conservation. Conserv. Biol. 16, 1048–1062 (2002).

[b35] Barnett L. A. K., Winton M. V., Ainsley S. M., Cailliet G. M. & Ebert D. A. Comparative Demography of Skates: Life-History Correlates of Productivity and Implications for Management. PLoS ONE 8, e65000. 10.1371/journal.pone.0065000. (2013). 23741442 PMC3669027

[b36] Fisher R. A., Call G. C. & Grubbs R. D. Age, Growth, and Reproductive Biology of Cownose rays (*Rhinoptera bonasus*) in Chesapeake Bay. Mar. Coast. Fish. 5, 224–235 (2013).

[b37] Frisk M. G., Miller T. J. Martell S. J. D. & Sosebee K. New hypothesis helps explain elasmobranch “outburst” on Georges Bank in the 1980 s. Ecol. Appl. 18, 234–245 (2008). 18372569 10.1890/06-1392.1

[b38] Castro J. I. Biology of the blacktip shark, *Carcharhinus limbatus*, off the southeastern United States. Bull. Mar. Sci. 59, 508–22 (1996).

[b39] Ellis J. K. & Musick J. A. Ontogenetic changes in the diet of sandbar shark, *Carcharhinus plumbeus*, in lower Chesapeake Bay and Virginia (USA) coastal waters. Environ. Biol. Fish. 80, 51–67 (2006).

[b40] Gelsleichter J., Musick J. A. & Nichols S. Food habits of the smooth dogfish, *Mustelus canis*, dusky shark, *Carcharhinus obscurus*, Atlantic sharpnose shark, *Rhizoprionodon terraenovae*, and the sand tiger, *Carcharias taurus*, from the northwest Atlantic Ocean. Environ. Biol. Fish. 54, 205–217 (1999).

[b41] Stillwell C. E. & Kohler N. E. Food habits of the sandbar shark *Carcharhinus plumbeus* off the U.S. northeast coast, with estimates of daily ration. Fish. Bull. 91, 138–150 (1993).

[b42] Arendt M. D., Olney J. E. & Lucy J. A., Stomach content analysis of cobia, *Rachycentron canadum*, from lower Chesapeake Bay. Fish. Bull. 99, 665–670 (2001).

[b43] Peterson C. H. & Summerson H. C. Basin-scale coherence of population dynamics of an exploited marine invertebrate, the bay scallop: implications of recruitment limitation. Mar. Ecol. Prog. Ser. 90, 257–272 (1992).

[b44] Mackenzie C. L. The bay scallop, *Argopecten irradians*, Massachusetts through North Carolina: its biology and the history of its habitats and fisheries. Mar. Fish. Rev. 70, 5–79 (2008).

[b45] Kirby M. X. Fishing down the coast: Historical expansion and collapse of oyster fisheries along continental margins. PNAS 101, 13096–13099 (2004). 15326294 10.1073/pnas.0405150101 PMC516522

[b46] Jackson J. B. C. *et al.* Historical overfishing and the recent collapse of coastal ecosystems. Science 293, 629–637 (2001). 11474098 10.1126/science.1059199

[b47] Wilberg M. J., Livings M. E., Barkman J. S., Morris B. T. & Robinson J. M. Overfishing, disease, habitat loss, and potential extirpation of oysters in upper Chesapeake Bay. Mar. Ecol. Prog. Ser. 436, 131–144 (2011).

[b48] Peterson C. H., Frodrie F. J., Summerson H. C. & Powers S. P. Site-specific and density-dependent extinction of prey by schooling rays: generation of apopulation sink in top-quality habitat for bay scallops. Oecologia 129, 349–356 (2001). 10.1007/s004420100742 28547190

[b49] Peterson C. H., Summerson H. C., Fegley S. R. & Prescott R. C. Timing, intensity and sources of autumn mortality of adult bay scallops *Argopecten irradians concentricus* Say. J. Exp. Mar. Biol. Ecol. 127, 121–140 (1989).

[b50] Prescott R. C. Sources of predatory mortality in the bay scallop *Argopecten irradians* (Lamarck): interactions with sea grass and epibiotic coverage. J. Exp. Mar. Biol. Ecol. 144, 63–83 (1990).

[b51] Tettelbach S. T. & Wenczel P. Reseeding efforts and the status of bay scallop *Argopecten irradians* (Lamarck, 1819) populations in New York following the occurrence of “brown tide” algal blooms. J. Shellfish Res. 12, 423–431 (1993).

[b52] Bricelj V. M. & Lonsdal D. J. *Aureococcus anophagefferens*: Causes and ecological consequences of brown tides in U.S. Mid-Atlantic coastal water. Limnol. Oceanogr. 42, 1023–1038 (1997).

[b53] Summerson H. C. & Peterson C. H. Recruitment failure of the bay scallop, *Argopecten irradians concentricus*, during the first red tide, *Ptychodiscus brevis*, outbreak recorded in North Carolina. Estuaries 13, 322–331 (1990).

[b54] Tester P. A., Stumpf R. P., Vukovich F. M., Fowler P. K. & Turner J. T. An expatriate red tide bloom: transport, distribution, and persistence. Limnol. Oceanogr. 36, 1053–1061 (1991).

[b55] Peterson C. H., Summerson H. C. & Luettich R. A. Jr. Response of bay scallops to spawner transplants: a test of recruitment limitation. Mar. Ecol. Prog. Ser. 132, 93–107 (1996).

[b56] Smith J. W. & Merriner J. V. Food habits and feeding behavior of the cownose ray, Rhinoptera bonasus, in lower Chesapeake Bay. Estuaries 8, 305–310 (1985).

[b57] Collins A. B., Heupel M. R., Hueter R. E. & Motta P. J. Hard prey specialists or opportunistic generalists? An examination of the diet of the cownose ray *Rhinoptera bonasus*. Mar. Freshw. Res. 58, 135–144 (2007).

[b58] Ajemian M. J. & Powers S. P. Habitat-specific feeding in cownose rays *Rhinoptera bonasus* of the northern Gulf of Mexico. Environ. Biol. Fish. 95, 79–97 (2012).

[b59] Fisher R. A. Life history, trophic ecology, and prey handling by Cownose Ray, *Rhinoptera bonasus*, from Chesapeake Bay. Virginia Institute of Marine Science, Virginia Sea Grant, Report 2010-20, VSG-10-25, Gloucester Point. (2010).

[b60] Fisher R. A., Call G. C. & Grubbs R. D. Cownose ray (*Rhinoptera bonasus*) predation relative to bivalve ontogeny. J. Shellfish Research 30, 187–196 (2011). Supplementary Materials References and Notes

[b61] Lo N. C., Jacobson L. D. & Squire J. L. Indices of relative abundance from fish spotter data based on delta lognormal models. Can. J. Fish. Aquat. Sci. 49, 2515–2516 (1992).

[b62] Maunder M. & Punt A. Standardizing catch and effort data: a review of recent approaches. Fish. Res. 70, 141–159 (2004).

[b63] Ortiz M. & Arocha F. Alternative error distribution models for standardization of catch rates of non-target species from a pelagic longline fishery: billfish species in the Venezuelan tuna longline fishery. Fish. Res. 70, 275–294 (2004).

[b64] Cooke J. G. & Lankester K. “Consideration of statistical models for catch-effort indices for use in tuning VPAs” (ICCAT Collective Volume of Scientific Papers 45, Madrid, Spain, 1996).

[b65] Stefansson G. Analysis of groundfish survey abundance data: combining the GLM and delta approaches. ICES J. Mar. Sci. 53, 577–588 (1996).

[b66] Caswell H. Matrix population models: construction, analysis and interpretation. (Sinauer Associates, Sunderland M. A., ed. 2, 2006).

[b67] Cortés E. Chondrichthyan demographic modeling: an essay on its use, abuse and future. Mar. Freshw. Res. 58, 4–6 (2007).

[b68] Hoenig J. M. Empirical use of longevity data to estimate mortality rates. Fish. Bull. 82, 898–903 (1983).

[b69] Pauly D. On the interrelationships between natural mortality, growth parameters, and mean environmental temperature in 175 fish stocks. Journal du Conseil 39, 175–192 (1980).

[b70] Peterson I. & Wroblewski J. S. Mortality rate of fishes in the pelagic ecosystem. Can. J. Fish. Aquat. Sci. 41, 1117–1120 (1984).

[b71] Chen S. & Watanabe S. Age dependence of natural mortality coefficient in fish population dynamics. Bull. Jap. Soc. Sci. Fish. 55, 205–208 (1989).

[b72] Fitz E. S. & Daiber F. C. An introduction to the biology of *Raja eglanteria* Bosc 1802 and *Raja erinacea* Mitchill 1825 as they occur in Delaware Bay. B. Bingham. Oceanogr. C. Collection 18, 69–97 (1963).

[b73] Packer D. B., Zetlin C. A. & Vitaliano J. J. Essential fish habitat source document: Little skate, *Leucoraja erinacea*, life history and habitat characteristics. NOAA Tech Memo NMFS NE 175, 66 pp. (2003).

[b74] McEachran J. D. & Musick J. A. Distribution and relative abundance of seven species of skates (Pisces: Rajidae) which occur between Nova Scotia and Cape Hatteras. Fish. Bull. 73, 110–136 (1975).

[b75] Able K. W. & Flescher D. Distribution and habitat of chain dogfish, *Scyliorhinus retifer*, in the Mid-Atlantic Bight. Copeia 1, 231–234 (1991).

[b76] Compagno L. J. V. 1984. FAO species catalogue. Vol. 4. Sharks of the world. An annotated and illustrated catalogue of shark species known to date. Part 2. Carchariniformes. FAO Fisheries Synopsis, No. 125, Volume 4, Part 2, p 251–655.

