# Supplementary material for: Critical assessment and ramifications of a purported marine trophic cascade
Source: Sci Rep. 2016 Feb 15;6:20970. doi: 10.1038/srep20970 (PMC4753470; doi:10.1038/srep20970)
Supplement: Supplemental Figures 1 and 2 [file srep20970-s1.pdf]

Supplemental information

Critical assessment and ramifications of a purported marine trophic cascade

R. Dean Grubbs , John K. Carlson, Jason G. Romine, Tobey H. Curtis, W. David McElroy, Camilla T. McCandless, Charles F. Cotton, John A. Musick

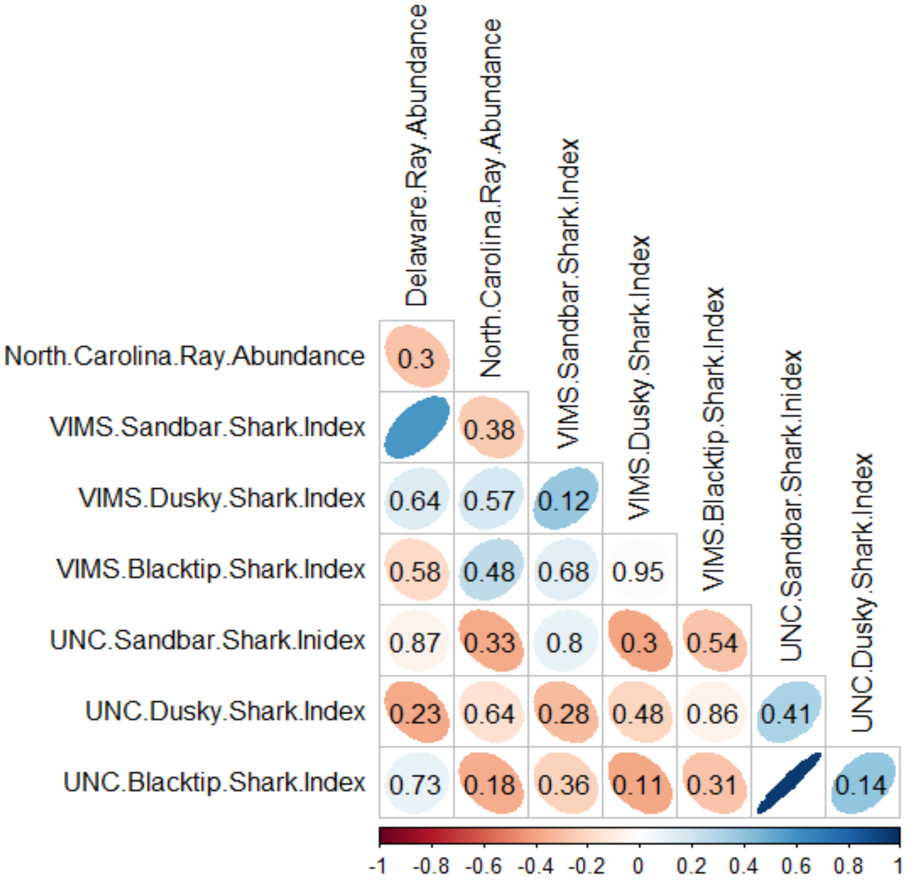

Supplemental Figure 1. Correlation matrix of model estimates for VIMS and UNC shark relative abundance (1990-2009) and Delaware Bay and North Carolina Cownose Ray relative abundance (1990-2004). Numerical values denote p-values for non-significant correlations (α= 0.05).

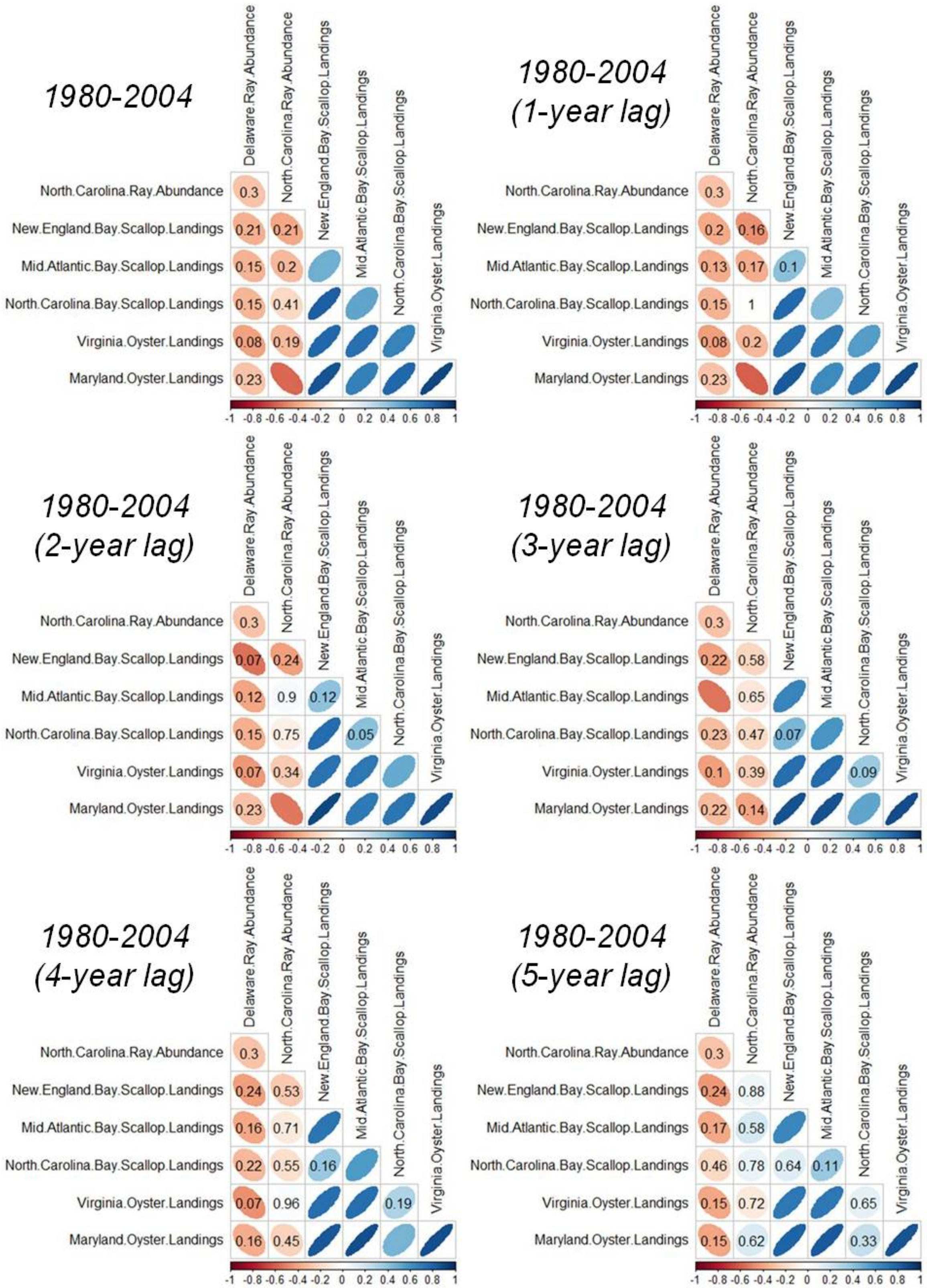

Supplemental Figure 2. Correlation matrices of model estimates for Delaware Bay and North Carolina cownose ray relative abundance and shellfish landings data including a 1,2,3,4, and 5-year lag of landings data for bay scallops (New England, Mid-Atlantic, and North Carolina) and oysters (Virginia and Maryland) between 1980 and 2004. Numerical values denote p-values for non-significant correlations ($\alpha = 0.05$).
